# Supplementary figures and images for: Loss-of-Function ROX1 Mutations Suppress the Fluconazole Susceptibility of upc2AΔ Mutation in Candida glabrata, Implicating Additional Positive Regulators of Ergosterol Biosynthesis
Source: mSphere. 2021 Dec 22;6(6):e00830-21. doi: 10.1128/msphere.00830-21 (PMC8694151; doi:10.1128/msphere.00830-21)

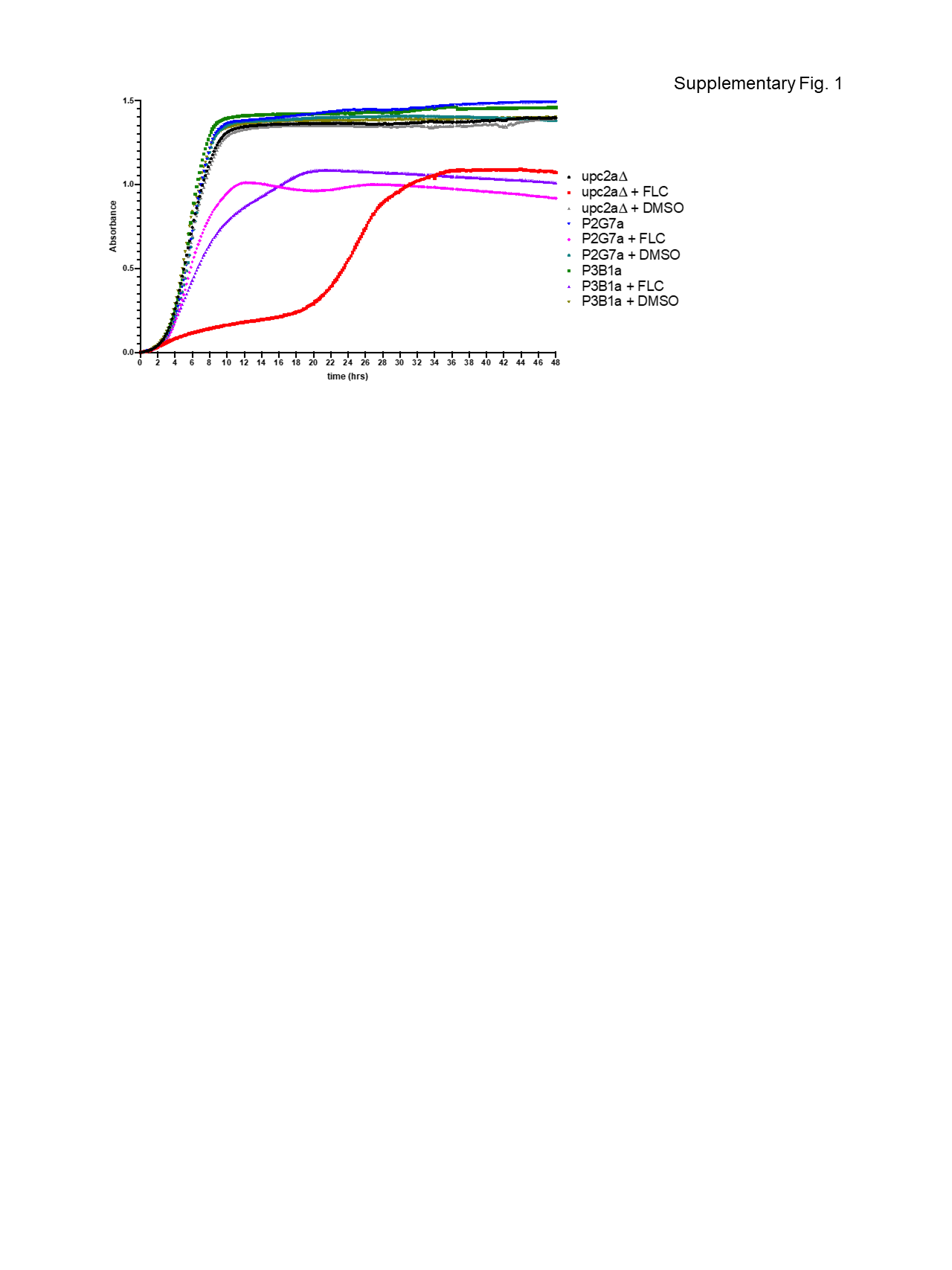

Supplement: FIG S1 [file msphere.00830-21-sf001.tif]
